# Supplementary material for: Chlamydia trachomatis-containing vacuole serves as deubiquitination platform to stabilize Mcl-1 and to interfere with host defense
Source: eLife. 2017 Mar 28;6:e21465. doi: 10.7554/eLife.21465 (PMC5370187; doi:10.7554/eLife.21465)
Supplement: Supplementary file 1. — X-ray crystallographic data collection and refinement statistics for the structure of Cdu1 (155-401). Data for the highest resolution shell are given in parentheses. a: Firedel’s mates were kept separately for calculation. DOI: http://dx.doi.org/10.7554/eLife.21465.034 [file elife-21465-supp1.docx]

**Supplementary File 1:** Data collection and refinement statistics

| **Data Collection** |  |  |
| --- | --- | --- |
| Crystal | **Native** | **SeMet Peak** |
| Space Group | P2_1_ | P2_1_ |
| Wavelength (Å) | 0.9184 | 0.9795 |
| Unit Cell Parameters  a, b, c (Å)  ß (°) | 39.10, 77.57, 68.95  96.43 | 39.74, 78.28, 69.32  96.19 |
| Resolution Limits (Å) | 38.85 – 1.70 (1-73 – 1.70) | 78.31 – 1.80 (1.84 – 1.80) |
| *R*_pim_ (%) | 6.4 (46.1) | 6.3 (50.3)^a^ |
| CC_1/2_ (%) | 99.7 (68.5) | 99.7 (79.0) |
| Observed Reflections | 151957 (7890) | 468220 (26965) |
| Unique Reflections | 44925 (2330) | 39189 (2292) |
| Multiplicity | 3.4 (3.4) | 11.9 (11.8) |
| Completeness | 99.8 (99.9) | 100 (99.9) |
| <*I/σI*> | 10.2 (1.8) | 12.6 (2.6) |
| **Phase Determination** |  |  |
| Anomalous Multiplicity |  | 6.0 (5.9) |
| Anomalous Completeness |  | 99.8 (99.8) |
| No. of sites |  | 11 |
| FOM after density modification  (PARROT) |  | 0.677 |
| **Refinement** |  |  |
| R_work_ (%) | 16.5 |  |
| R_free_ (%) | 21.3 |  |
| RMSD Bond Lengths (Å) | 0.006 |  |
| RMSD Bond Angles (°) | 0.941 |  |
| Ramachandran statistics (%) | 98 /1.8/0.2 |  |
| Data for the highest resolution shell are given in parentheses  ^a^: Friedel's mates were kept separately for calculation | | |
