# Supplementary material for: Chlamydia trachomatis-containing vacuole serves as deubiquitination platform to stabilize Mcl-1 and to interfere with host defense
Source: eLife. 2017 Mar 28;6:e21465. doi: 10.7554/eLife.21465 (PMC5370187; doi:10.7554/eLife.21465)
Supplement: Supplementary file 2. — Listed are all plasmids used in this study to transfect human cells or to transform E. coli or C. trachomatis. If not stated otherwise, constructs were cloned in this study using the oligo nucleotides listed in Supplementary file 4. DOI: http://dx.doi.org/10.7554/eLife.21465.035 [file elife-21465-supp2.docx]

**Supplementary File L2:** Plasmids

| Name | Insert | Backbone | origin |
| --- | --- | --- | --- |
| pCDNA3/HA-ubiquitin | HA-ubiquitin | pCDNA3 | Dirk Bohmann |
| pCDNA3.1/hMcl-1 | hMcl-1 | pCDNA3.1 | Roger Davis;  addgene plasmid 25375 |
| pCDNA3/myc-Mcl-1 | myc-Mcl-1 | pCDNA3 | This work |
| pEGFP | GFP | pEGFP-N1 |  |
| pEGFP/Mcl-1 | Mcl-1-GFP | pEGFP-N1 | This work |
| pGFP::SW2 |  |  | Ian Clarke |
| pGEX4t3/ΔNHECTH9 | ΔNHECTH9 | pGEX4t3 | Martin Eilers |
| pCDNA3/Cdu1 | Cdu1-FLAG | pCDNA3 | This work |
| pCDNA3/Cdu1(C345A)-FLAG | Cdu1(C345A)-FLAG | pCDNA3 | This work |
| pET28a/Cdu1 | His-Cdu1 | pET28a | This work |
| pET28a/GroEL | His-GroEL | pET28a | This work |
| pET28a/Cdu2 | His-Cdu2 | pET28a | This work |
| pGEX4t3/Mcl-1 | GST-Mcl-1 | pGEX4t3 | This work |
| pAH1 |  |  | This work |
| pAH3 |  |  | This work |
| pTet::SW2 |  | pSW2 | This work, inducible protein expression in *Ctr* |
| pTet/Cdu1-FLAG::SW2 |  | pSW2 | This work |
| pTet/Cdu2-FLAG::SW2 |  | pSW2 | This work |
