# Supplementary material for: Chlamydia trachomatis-containing vacuole serves as deubiquitination platform to stabilize Mcl-1 and to interfere with host defense
Source: eLife. 2017 Mar 28;6:e21465. doi: 10.7554/eLife.21465 (PMC5370187; doi:10.7554/eLife.21465)
Supplement: Supplementary file 3. — Listed are all C. trachomatis strains used and generated in this study. All C. trachomatis strains generated by transformation and selection originate from the C. trachomatis LGV L2 (434) (ATCC VR-902B) strain. DOI: http://dx.doi.org/10.7554/eLife.21465.036 [file elife-21465-supp3.docx]

**Supplementary File 3:** *Chlamydia* *trachomatis* strains

| Species | Serovar | Properties | Source |
| --- | --- | --- | --- |
| *C. trachomatis* | LGV L2 (434) |  | ATCC^®^ VR-902B^™^ |
| *C. trachomatis* pGFP::SW2 | LGV L2 (434) | *C. trachomatis* transformed with the pGFP::SW2 plasmid. Selection with 50 U Peng/ml | Adrian Mehlitz |
| *C. trachomatis* Cdu1-FLAG | LGV L2 (434) | *C. trachomatis* transformed with the pAH1 plasmid. Selection with 5 U PenG/ml. | this work |
| *C. trachomatis* pTet/Cdu1-FLAG::SW2 | LGV L2 (434) | *C. trachomatis* transformed with pTet/Cdu1-FLAG::SW2 plasmid. Selection with 10 U PenG/ml. Induction with 10 ng/ml AHT. | This work |
| *C. trachomatis* pTet/Cdu2-FLAG::SW2 | LGV L2 (434) | *C. trachomatis* transformed with pTet/Cdu2-FLAG::SW2 plasmid. Selection with 10 U PenG/ml. Induction with 10 ng/ml AHT. | This work |
| *C. trachomatis* pIncA-FLAG::SW2 | LGV L2 (434) | *C. trachomatis* transformed with pIncA-FLAG::SW2 plasmid. Selection with 10 U PenG/ml. | Prema Subbarayal |
| *C. trachomatis* Tn*-cdu1* | LGV L2 (434) | *C. trachomatis* transposon insertion mutant | Harrison et al. |
| *C. trachomatis* Tn*-*IGR | LGV L2 (434) | *C. trachomatis* transposon insertion mutant | Harrison et al. |
