# Supplementary material for: Chlamydia trachomatis-containing vacuole serves as deubiquitination platform to stabilize Mcl-1 and to interfere with host defense
Source: eLife. 2017 Mar 28;6:e21465. doi: 10.7554/eLife.21465 (PMC5370187; doi:10.7554/eLife.21465)
Supplement: Supplementary file 4. — Listed are all oligo nucleotides in 5’ → 3’ orientation used in this study. The oligo nucleotides were used for construct cloning, sequencing or southern hybridization as indicated in the comment column. DOI: http://dx.doi.org/10.7554/eLife.21465.037 [file elife-21465-supp4.docx]

**Supplementary File 4:** Oligo nucleotides

| Name | Sequence 5’ 🡪 3’ | comment |
| --- | --- | --- |
| Cdu1 frw | CTGATGTTATCTCCCACCAACTCA | Cloning in pCDNA3-FLAG |
| Cdu1 rev | TCGTGCTTCAGGCCAAGAAAGCT | Cloning in pCDNA3-FLAG |
| Cdu1 Mut frw | GATCCAGCGCCGGCGCTT | Site directed mutagenesis |
| Cdu1 Mut rev | AAGCGCCGGCGCTGGATC | Site directed mutagenesis |
| Cdu1 frw II | TCGTTATCTCCCACCAACTCAACTT | Cloning in pET28a |
| Cdu1 rev II | CTGTTATGCTTCAGGCCAAGAAAGC | Cloning in pET28a |
| GroEL frw | TCGGTCGCTAAAAACATTAAATACAAC | Cloning in pET28a |
| GroEL rev | CTGTTAGAGTTGACCGCCAGTTAAGA | Cloning in pET28a |
| Cdu2 frw | TCGGAACCAATTCATAATCCTCCCC | Cloning in pET28a |
| Cdu2 rev | CTGTTAATCCGTAGTTGGCCAGCT | Cloning in pET28a |
| Mcl-1 frw | GATCGGATCCTTTGGCCTCAAAAGAAACGCG | Cloning in pGEX4t3 |
| Mcl-1 rev | GATCGTCGACCTATCTTATTAGATATGCCAAAC | Cloning in pGEX4t3 |
| P1 pAH1 | GATCACTAGTATGTTATCTCCCAC | CTL0247 part for pAH1 |
| P2 pAH1 | ATGCAGCTCCCGGAATTCTCACCAATAA |  |
| P3 pAH1 | TTATTGGTGAGAATTCCGGGAGCTGCAT | Selection cassette for pAH1 |
| P4 pAH1 | GATTAGAAAAGAGTTTACGCCCCGCCCT |  |
| P5 pAH1 | AGGGCGGGGCGTAAACTCTTTTCTAATC | 3’ region from CTL0247 for pAH1 |
| P6 pAH1 | GATCACTAGTTCTTCTTGATCGGA |  |
| P1 pAH3 | GATGCTGTTTTACGAGGGGAGC | Opened pQE30 backbone for DiTriSec insert integration |
| P2 pAH3 | CGTCTAAAATCGAGATGATTTTCTGT |  |
| P3 pAH3 | GATCATCTGATAATGGTTTCTTAGA | Generation of selection cassette for pAH3 |
| P4 pAH3 | GATCGTCGACCATTAATCTAGATA |  |
| Seq. pAH1 frw | CCTTATCCTCTTCCCGTCTA | Sequencing of *C. trachomatis* Cdu1-FLAG for integration of the pAH1 plasmid |
| pTet Cdu1 frw | AGCATGTTATCTCCCACCAACTCA | Cloning in pTet::SW2 |
| pTet Cdu1 rev | GACTTACTTATCGTCGTCATCCTTGTAATC |  |
| pTet Cdu2 frw | AGCATGGAACCAATTCATAATCCTCCCCC | Cloning in pTet::SW2 |
| pTet Cdu2 rev | GACTTACTTATCGTCGTCATCCTTGTAATCATCCGTAGTTGGCCAG |  |
| Fw:2 | GATCACGCGTCGGAAATTTGGTTATC | Cloning of IncA-FLAG in pGFP::SW2 |
| Rv:2A | CGATTAGAGTAGGCGTTGTCATATCGGCTTCCTTTTGTAAATTTGATA |  |
| Fw:3A | TATCAAATTTACAAAAGGAAGCCGATATGACAACGCCTACTCTAATCG |  |
| Rv:3A | GATCGTCGACATTAATCTAGATATCGAGCTCGAGTTACTTATCGTCGTCATCCTTGTAATCagcgtaatctggaacatcgtatgggtaGGAGCTTTTTGTAGAGGGTGAT |  |
| bla frw | GATCATCTGATAATGGTTTCTTAGA | Amplification of Southern hybridization probe 1 |
| bla rev | CATAGGCTCCGCCCCTGACGCTCAGTGG |  |
| 5’ region frw | GATCACTAGTGGGGAATTACAGGA | Amplification of Southern hybridization probe 2 |
| 5’ region rev | ATGCAGCTCCCGGAAGGGCTATTTGCTT |  |
